# Evolution of the codling moth pheromone via an ancient gene duplication

**DOI:** 10.1186/s12915-021-01001-8

**Published:** 2021-04-23

**Authors:** Jean-Marc Lassance, Bao-Jian Ding, Christer Löfstedt

**Affiliations:** 1 Department of Biology, Lund University, Sölvegatan 37, SE-223 62 Lund, Sweden; 2 Department of Organismic and Evolutionary Biology, Harvard University, 16 Divinity Avenue, Cambridge, MA 02138 USA

**Keywords:** Fatty acyl desaturase, Gene family evolution, Bifunctional, Conjugated double bond, Tortricidae

## Abstract

**Background:**

Defining the origin of genetic novelty is central to our understanding of the evolution of novel traits. Diversification among fatty acid desaturase (FAD) genes has played a fundamental role in the introduction of structural variation in fatty acyl derivatives. Because of its central role in generating diversity in insect semiochemicals, the FAD gene family has become a model to study how gene family expansions can contribute to the evolution of lineage-specific innovations. Here we used the codling moth (*Cydia pomonella*) as a study system to decipher the proximate mechanism underlying the production of the ∆8∆10 signature structure of olethreutine moths. Biosynthesis of the codling moth sex pheromone, (*E*8,*E*10)-dodecadienol (codlemone), involves two consecutive desaturation steps, the first of which is unusual in that it generates an *E*9 unsaturation. The second step is also atypical: it generates a conjugated diene system from the *E*9 monoene C_12_ intermediate via 1,4-desaturation.

**Results:**

Here we describe the characterization of the FAD gene acting in codlemone biosynthesis. We identify 27 FAD genes corresponding to the various functional classes identified in insects and Lepidoptera. These genes are distributed across the *C. pomonella* genome in tandem arrays or isolated genes, indicating that the FAD repertoire consists of both ancient and recent duplications and expansions. Using transcriptomics, we show large divergence in expression domains: some genes appear ubiquitously expressed across tissue and developmental stages; others appear more restricted in their expression pattern. Functional assays using heterologous expression systems reveal that one gene, Cpo_CPRQ, which is prominently and exclusively expressed in the female pheromone gland, encodes an FAD that possesses both *E*9 and ∆8∆10 desaturation activities. Phylogenetically, Cpo_CPRQ clusters within the Lepidoptera-specific ∆10/∆11 clade of FADs, a classic reservoir of unusual desaturase activities in moths.

**Conclusions:**

Our integrative approach shows that the evolution of the signature pheromone structure of olethreutine moths relied on a gene belonging to an ancient gene expansion. Members of other expanded FAD subfamilies do not appear to play a role in chemical communication. This advises for caution when postulating the consequences of lineage-specific expansions based on genomics alone.

## Background

Establishing the origin of genetic novelty and innovation is central to our understanding of the evolution of novel traits. While genes can evolve de novo in the genome, the most common mechanism involves duplications of existing genes and the subsequent evolution of novel properties harbored by the encoded gene products. Expansions provide opportunities for specialization and evolution of novel biological functions within a lineage, including the breadth of expression via modifications of promoter architecture [1]. The size of gene families is influenced by both stochastic processes and selection, and particularly large differences in genetic makeup can be indicative of lineage-specific adaptation and potentially associate with traits contributing to phenotypical differentiation between groups [2, 3]. However, the precise functional consequences of such amplifications remain frequently unclear, even if expansions show readily discernible patterns. Therefore, deciphering the underlying genetic and molecular architecture of new phenotypic characters is necessary for a complete understanding of the role played by the accumulation of genetic variation through gene duplication.

For organisms relying on chemical communication, evolution of the ability to produce and detect a new type of molecule could allow for the expansion of the breadth of available communication channels and provide a medium with no or limited interference from other broadcasters. Since the identification of bombykol by Butenandt and co-workers in 1959 [4], a countless number of studies have contributed to revealing the diversity of fatty acid derivatives that play a pivotal role in the chemical communication of insects. As a consequence of homologies with well-studied metabolic pathways, our understanding of the molecular basis of pheromone biosynthesis from fatty acid intermediates has greatly advanced over the past two decades, highlighting the role of several multigene families [5]. Insect semiochemicals are synthesized in specialized cells in which fatty acyl intermediates are converted in a stepwise fashion by a combination of desaturation, chain-shortening, and chain-elongation reactions followed by modifications of the carbonyl group, to cite a few possible steps ([5, 6] and references therein). Desaturation appears particularly important and contributes to producing the great diversity of structures observed in insect pheromones. This derives from the properties of the enzymes that are central to many uncanonical fatty acid synthesis pathways seen in insects. These enzymes can exhibit diverse substrate preference, introduce desaturation in either or both *cis* (*Z*) and *trans* (*E*) geometry, and give rise to variation in chain-length double-bond position, number, and configuration.

In insects, the group of proteins responsible for catalyzing desaturation reactions are fatty acyl-CoA desaturases (FADs). These membrane-bound acyl-lipid desaturases are biochemically and structurally homologous to the desaturases ubiquitously found in animals, yeast, fungi, and many bacteria where they play important basic biological functions in lipid metabolism and cell signaling and contribute to membrane fluidity in response to temperature fluctuation [7]. In the past decades, the integration of molecular and phylogenetic approaches has greatly advanced our understanding of the function of FAD genes in the biosynthesis of mono- and poly-unsaturated fatty acids in a range of organisms. Moreover, an ever-increasing number of acyl-CoA desaturase genes have been functionally characterized, demonstrating mechanistically their crucial role in the biosynthesis of pheromone and semiochemicals in *Drosophila* fruitflies, bees, wasps, beetles, and lepidopteran species. Variation in the number and expression of acyl-CoA desaturase genes have been shown to affect the diversity of pheromone signals between closely related species [8–12]. The family is characterized by multiple episodes of expansion and contraction that occurred during the evolution of insects [13, 14]. Consequently, the FAD gene family has become a model to study how structural and regulatory changes act in concert to produce new phenotypes.

**Fig. 1:**
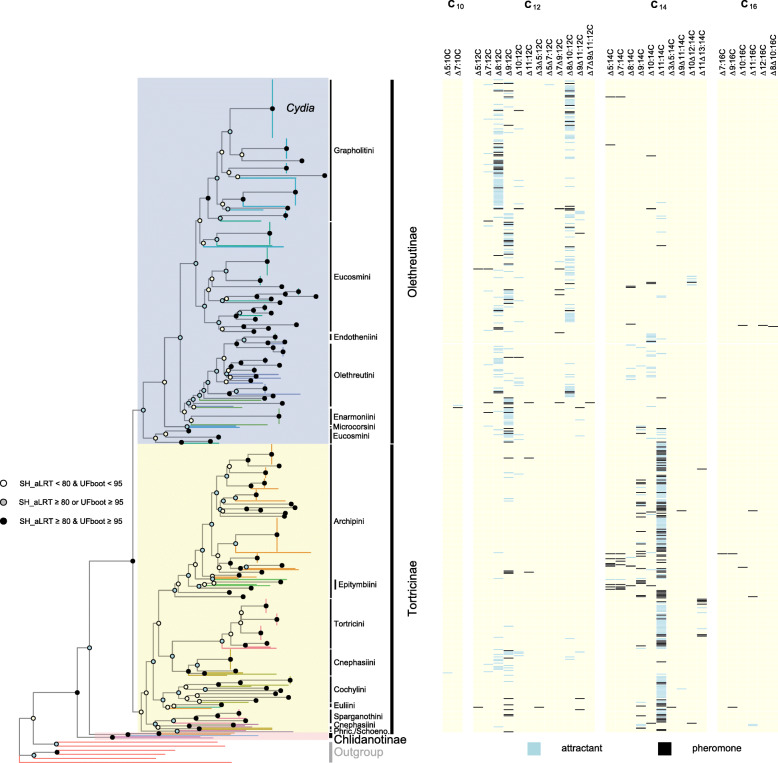
Phylogeny of Tortricidae and their associated female sex pheromone components. (left) The maximum likelihood tree was obtained for predicted nucleotide sequences of Tortricidae species (7591 aligned positions). The species represented comprise all tortricids for which pheromones or attractants have been reported plus some outgroups. Typically, one representative species was chosen per genus and contributed molecular evidence for all species in the genus. Outgroup species are represented by red branches whereas tortricid species from the same tribe are represented by branches of the same color. Branch support values were calculated from 1000 replicates using the Shimodaira-Hasegawa-like approximate ratio test (SH_aLRT) and ultrafast bootstrapping (UFboot). Support values for branches are indicated by colored circles, with color assigned based on thresholds of branch selection for SH-aLRT (80%) and UFBoot (95%) supports, respectively. The major subfamilies and represented tribe names are indicated (Phric: Phricanthini; Schoeno: Schoenotenini). (right) Heatmap representing the presence/absence of unsaturated fatty acid structure in bioactive molecules. Attractants correspond to compounds found to be attractive in either field or laboratory experiments; pheromone components correspond to sex attractants produced naturally by the organism and with a demonstrated biological activity on conspecific males. Double-bond positions are annotated in Δ-nomenclature without referring to the geometry. Molecular and trait data retrieved from GenBank and the Pherobase, respectively

Among the taxa available to study the molecular basis of pheromone production in an evolutionary framework, leafroller moths (Lepidoptera: Tortricidae) provide a model system of choice [15]. Leafrollers represent one of the largest families in the Lepidoptera with over 10,000 described species [16]. Their larvae feed as leaf rollers, leaf webbers, leaf miners, or borers in plant stems, roots, fruits, or seeds. Many tortricid species are important pests and, due to their economic impact on human society, became the target of many pheromone identification studies. In 1982, Roelofs and Brown published a comprehensive review on pheromones and evolutionary relationships among Tortricidae [17]. These authors related the patterns in pheromone diversity in a biosynthetic perspective to different postulated phylogenies of the Tortricidae, and specifically the two major groups within Tortricidae, Tortricinae and Olethreutinae. Based on the pheromone identifications available at the time, it was suggested that species in the Tortricinae use mostly 14-carbon pheromone components (acetates, alcohols, and aldehydes) whereas species in the Olethreutinae subfamily use mostly 12-carbon compounds. Building on recent advances towards a robust molecular phylogeny of Tortricidae [18, 19] and incorporating the information for the pheromones identified in 179 species and sex attractants reported for an additional 357 species, we show that the proposed dichotomy is well-supported by the data currently available (Fig. 1). Furthermore, a majority of species in Tortricinae use pheromone components with double bonds in uneven positions, i.e., ∆9 isomers or ∆11 isomers. By contrast, the Olethreutinae pheromones typically contain components with double bonds in even positions (∆8, ∆10), with doubly unsaturated ∆8∆10:12C fatty acyl chains being one of the signature structures of the subfamily. Roelofs and Brown [17] suggested that the use of a Lepidoptera-specific ∆11-desaturase acting on myristic acid (C_14_) could account biosynthetically for most of the pheromone compounds found in Tortricinae. This hypothesis was later confirmed with the functional characterization of FADs expressed in the female pheromone gland of several Tortricinae representatives. These include a desaturase that makes only the *E*11-isomer in the light brown apple moth *Epiphyas postvittana* (E11-14, E11-16, and E9E11-14) [20], as well as desaturases from the redbanded leafroller moth *Agryrotaenia velutinana* [21] and the obliquebanded leafroller moth *Choristoneura rosaceana* [22] which both produce a mixture of Z/E11-14:Acids. In the case of Olethreutinae, ∆11 desaturation followed by chain-shortening could account for the ∆9:12C compounds found in several tribes of Olethreutinae. On the other hand, the biochemical pathways leading to the pheromone components with double bonds in even positions, the ∆8 and ∆10 as well as the doubly unsaturated ∆8∆10:12C compounds in the Olethreutinae, were not obvious.

**Fig. 2:**
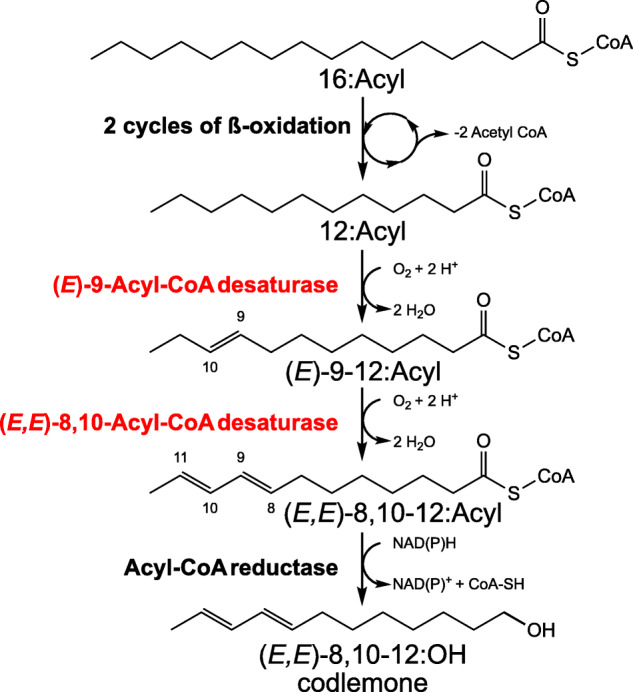
Pathway of codlemone biosynthesis. Palmitic acyl (C_16_) is first formed by the fatty acid synthesis pathway. Lauric acyl (C_12_) is then produced following two recurring reactions of beta-oxidation. A first desaturation occurs, introducing an *E*9 double bond between carbons 9 and 10 from the carboxyl terminus. The resulting monoenoic fatty acyl is then the substrate of a second desaturation via the removal of hydrogen atoms at the carbons 8 and 11, causing the formation of a *E*8*E1*0 conjugated diene system. Finally, an unknown fatty acyl-CoA reductase converts the fatty acyl precursor into the fatty alcohol (*E*8,*E*10)-8,10-dodecadienol commonly known as codlemone. The steps characterized in this study are highlighted in red

The codling moth *Cydia pomonella* (Linnaeus) (Tortricidae: Olethreutinae: Grapholitini) is one of the most devastating pests in apple and pear orchards worldwide [23]. With the goal of disrupting its reproduction, it has received substantial attention and been at the center of numerous studies focused on characterizing its communication system via sex pheromones. Its pheromone, (*E*8,*E*10)-dodecadien-1-ol, also known under the common name *codlemone*, was first identified using gas chromatography in combination with electroantennogram (EAG) recordings [24]. This identification was later confirmed by fine chemical analysis [25, 26]. The codling moth thus provides a relevant system to unravel the molecular pathway associated with the production of the ∆8∆10:12C typical of olethreutines. Previous studies support the hypothesis that the biosynthesis involves the desaturation of a ∆9 monoene intermediate. First, Arn et al. [27] reported the presence of the unusual (*E*)-9-dodecenol (E9-12:OH) at about 10% of the doubly unsaturated alcohol in pheromone gland extracts and effluvia from *C. pomonella* females. Although this monoene does not carry any behavioral activity, its occurrence suggested that E9-12:Acyl could be an intermediate of codlemone biosynthesis in a process analogous to the biosynthesis of 10,12-dienic systems via ∆11 monoene intermediates observed in *Bombyx mori*, *Manduca sexta*, and *Spodoptera littoralis* [28–30]. The selective incorporation of deuterium-labeled E9-12 fatty acid precursors into codlemone and its direct precursor, *E*8,*E*10-dodecadienoate (E8E10-12:Acyl), supported the presence of an unusual ∆9 desaturase in *C. pomonella* and the biosynthesis of a conjugated diene system via 1,4-desaturation and the characteristic elimination of two hydrogen atoms at the allylic position of the double bond in the monoene intermediate precursor [31] (Fig. 2). Similar conclusions were reached from a replicate study using *C. splendana* and *C. nigricana* females [32]. To date, the FAD(s) central to the biosynthesis of codlemone and related fatty acyl derivates with a ∆8∆10 system has not been characterized.

The availability of a high-quality draft genome for *C. pomonella* provides an opportunity to comprehensively annotate and analyze relevant genes [33]. Here we annotated a total of 27 FAD genes and performed a phylogenetic analysis, revealing expansions of different ages in the ∆9(KPSE) clade and in the ∆10/∆11(XXXQ/E) clade. Using a transcriptomic approach, we determine the breadth of expression of all FAD genes and identified genes with upregulated expression in the pheromone gland of female *C. pomonella*, where the biosynthesis of codlemone takes place. We tested the function of these desaturases in heterologous expression systems and identified one FAD gene conferring on the cells the dual desaturase functions playing a key role in the biosynthesis of (*E8*,*E*10)-dodecadienol in *C. pomonella* and a signature structure of many Olethreutinae moth pheromones.

## Results

### Expansion of FADs in the genome of *C. pomonella*

**Fig. 3:**
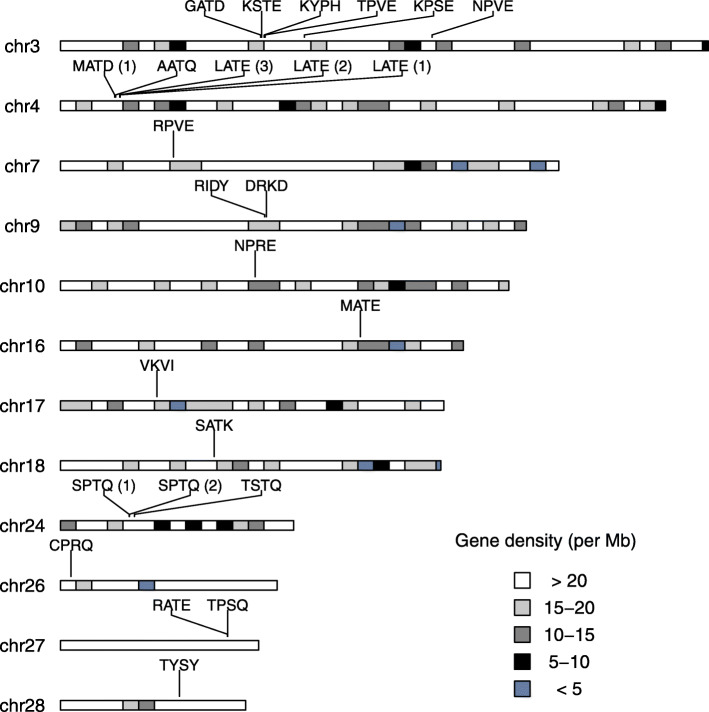
Genomic organization of fatty acyl desaturase genes in *Cydia pomonella*. The positions of the 27 predicted FAD genes were mapped to the genome. Twenty-five genes could be placed on 12 autosomes; 2 genes, Cpo_MATD(2) and Cpo_QPVE, are located on unplaced scaffolds (not drawn). The distribution analysis showed that there are 5 gene clusters which contain 2 or more FAD genes. Names refer to chromosome names in the genome assembly. Chromosomes are drawn to scale and the bands represent gene density, with darker bands depicting gene-poor regions

We identified candidates potentially involved in fatty acid synthesis by searching in the genome of *C. pomonella* for genes encoding fatty acid desaturases, which are characterized by a fatty acid desaturase type 1 domain (PFAM domain PF00487). First, we improved the genome annotation by incorporating data from the pheromone gland transcriptome, a tissue which was not part of the panel of tissues used to generate the available annotation of *C. pomonella*. Searching our improved annotation, we identified 27 genes harboring the signature domain of FADs. While the vast majority of genes identified in our exhaustive search contain open-reading frames of 300 amino acids or longer, a small number of genes (i.e., 2–3) may represent pseudogenes or assembly errors. We adopted the nomenclature proposed by Knipple et al. [34] in which genes are named based on the composition of 4 amino acid residues at a signature motif. Next, we looked at their genomic organization. We found that FAD genes are distributed across 12 of the 27 autosomes, with no FAD genes on either Z or W sex chromosome (chr1 and chr29, respectively) (Fig. 3). Two genes, Cpo_QPVE and Cpo_MATD(2), were found on unplaced scaffolds. As is typical for members of a multigene family, we identify several clusters corresponding to tandemly duplicated genes. Moreover, several genes are present as the single member of the family on a given autosome.

### Phylogenetic analyses place expansions in the ∆9(KPSE) and ∆10/∆11(XXXQ/E) clades

**Fig. 4:**
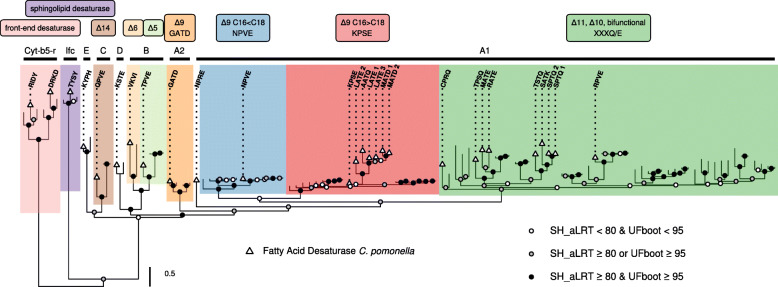
Phylogeny of Lepidoptera FAD genes. The maximum likelihood tree was obtained for the predicted amino acid sequence of 114 FAD genes (805 aligned positions) of 28 species, with branch support values calculated from 1000 replicates using the Shimodaira-Hasegawa-like approximate ratio test (SH_aLRT) and ultrafast bootstrapping (UFboot). Support values for branches are indicated by colored circles, with color assigned based on SH-aLRT and UFBoot supports using 80% and 95% as thresholds of branch selection for SH-aLRT and UFBoot supports, respectively. The major constituent six subfamilies of First Desaturase (A1 to E) and two subfamilies of Front-End (Cyt-b5-r) and Sphingolipid Desaturases (Ifc), respectively, are indicated following the nomenclature proposed by Helmkampf et al. [14]. For First Desaturases, the different shades correspond to the indicated putative biochemical activities and consensus signature motif (if any). Triangles indicate sequences from *C. pomonella* (see also Additional file 1: Figure S1 for the extended version of this tree). The scale bar represents 0.5 substitutions per amino acid position

In order to determine which of the genes identified above could be important for codlemone biosynthesis, we assessed the functional classes represented by these genes using phylogenetic analyses. To that end, we aligned the predicted protein sequences of the *C. pomonella* genes with a panel of FAD sequences from Lepidoptera species including genes for which function has been previously characterized using in vitro heterologous expression. We found representatives of the eight insect acyl-CoA desaturase subfamilies sensu Helmkampf et al. [14] (Fig. 4; Additional file 1: Figure S1).

All subfamilies form highly supported groups, with the exception of the relationship between Desat A1 and A2, which appear weakly or strongly supported, depending on the set of genes used in our analyses. With a characteristic domain architecture (PF08557 in front of PF00487), Cpo_TYSY encodes a putative Sphingolipid Delta-4 desaturase and is homologous to *interfertile crescent* in *D. melanogaster* (Ifc). Two tandemly duplicated genes, Cpo_RIDY and Cpo_DRKD, contain a Cytochrome b5-like heme binding domain (PF00173) in front of the fatty acid desaturase domain and group with putative Front-End desaturases homologous to *Cytochrome b5-related* in *D. melanogaster* (Cyt-b5-r). These three genes bear little similarity with the other 24 FAD genes which encode First Desaturases of the subfamilies Desat A1 through E.

Desat C, D, and E are present as single-copy genes in *C. pomonella* (Cpo_QPVE, Cpo_KSTE, and Cpo_KYPH, respectively). Cpo_QPVE groups with the ∆14 desaturase identified in male and female corn borer pheromone biosynthesis (Lepidoptera: Crambidae) [9, 35].

Desat B, which is particularly expanded in Hymenopterans and in *Bombyx mori* [14], has only two representatives in *C. pomonella*. These genes, Cpo_VKVI and Cpo_TPVE, are homologous to the *E*6 desaturase identified in *Antheraea pernyi* (Lepidoptera: Saturnidae) [36] and the *Z*5 desaturase identified in *Ctenopseustis obliquana* (Lepidoptera: Tortricidae) [37], respectively.

Desat A2 subfamily is characterized by a single-copy gene, Cpo_GATD, which seems to be typical of Lepidoptera. The homolog identified in *Choristoneura parallela* (Lepidoptera: Tortricidae) is associated with the formation of ∆9 desaturation in saturated acyl moieties of a range of length (C_14_-C_26_) [38].

Finally, Desat A1 forms the largest group and experienced a particularly dynamic evolutionary history. With 17 genes, *C. pomonella* harbors twice as many genes as *Bombyx mori* (7 in the most recent version of the genome SilkDB 3.0 [39]). While ∆9 C16<C18 (NPVE) is represented by a single-copy gene, we found two significant expansions in the group corresponding to ∆9 C16>C18 (KPSE) and ∆11, ∆10, and bifunctional (XXXQ/E) FADs. The tandemly arrayed cluster found in chr4 corresponds to an extension within the A1 subfamily encoding ∆9 C16>C18 FADs. Nine genes are found in the Lepidoptera-specific A1 subfamily encoding ∆11, ∆10, and bifunctional FADs. These genes are scattered across 6 private chromosomes, none of which containing members of the other functional classes.

Based on their placement in the FAD gene tree, these genes can be sub-divided into four groups (Fig. 4; Additional file 1: Figure S1). Group1 contains Cpo_SPTQ(1), Cpo_SPTQ(2), Cpo_SATK, and Cpo_TSTQ, which are homologous to the ∆10 desaturase identified in the New Zealand tortricid *Planotortrix octo* (Lepidoptera: Tortricidae) [40]. Group2 is formed with Cpo_RATE, Cpo_MATE, and Cpo_TPSQ, which are homologous to the FAD with ∆6 activity isolated from *Ctenopseustis herana* (Lepidoptera: Tortricidae) [12]. Group3 is represented by Cpo_RPVE, which groups with desat2 from *Ctenopseustis obliquana* and allied species (Lepidoptera: Tortricidae) [12] and Lca_KPVQ from *Lampronia capitella* (Lepidoptera: Prodoxidae) [41]. Finally, Cpo_CPRQ falls near Dpu_LPAE from *Dendrolimus punctatus* (Lepidoptera: Lasiocampidae) [42]. Based on our phylogenetic reconstructions, several genes are plausible candidates in the biosynthesis of codlemone. First, genes from the ∆9 C16>C18 (KPSE) subfamily have been implicated in pheromone biosynthesis and carry activities similar to those expected in *C. pomonella*. Specifically, the Dpu_KPSE from *Dendrolimus punctatus* (Lepidoptera: Lasiocampidae) produces a range of ∆9-monounsaturated products including Z9- and E9-12:Acyl [42]. This exemplifies that enzymes in this subfamily can introduce *cis* and *trans* double bonds in lauric acid (C_12_). Furthermore, when supplemented with Z7- and E7-14:Acyl, Dpu_KPSE can introduce a second desaturation to produce ∆7∆9-14:Acyl, illustrating the formation of conjugated double bonds by this type of FAD. The expansion of the KPSE clade we report in *C. pomonella* is an interesting coincidence. Second, the abundance of representatives in the ∆11, ∆10, and bifunctional (XXXQ/E) subfamily supports the possibility that one or several could be involved in codlemone biosynthesis. This group contains many genes involved in the biosynthesis of conjugated double bonds, e.g., Bmo_KATQ in *Bombyx mori* (Lepidoptera: Bombycidae) [43], Mse_APTQ in *Manduca sexta* (Lepidoptera: Sphingidae) [44], Lca_KPVQ in *Lampronia capitella* (Lepidoptera: Prodoxidae) [41], and Dpu_LPAE in *Dendrolimus punctatus* [42]. Cpo_RPVE groups with Lca_KPVQ, which encodes an enzyme involved in the desaturation of 16:Acyl and Z9-14:Acyl to produce the conjugated Z9Z11-14:Acyl pheromone precursor of *L. capitella* [41]. Another interesting candidate is Cpo_CPRQ, which clusters near Dpu_LPAE, an enzyme capable of catalyzing the production of E9Z11-16:Acyl and E9E11-16:Acyl [42].

### Patterns of gene expression identify Cpo_CPRQ and Cpo_SPTQ(1) as candidates

To identify candidates for codlemone production, we compared the expression levels of the twenty-seven genes found in the genome using published RNA sequencing (RNA-Seq) data from various tissues and life stages augmented with two new pheromone gland data sets (this study). Since codlemone is found in the pheromone gland of females, which is located at the tip of the abdomen, we hypothesized that its desaturase(s) would be enriched or even exclusively expressed in this sex and in the population of cells forming this tissue.

**Fig. 5:**
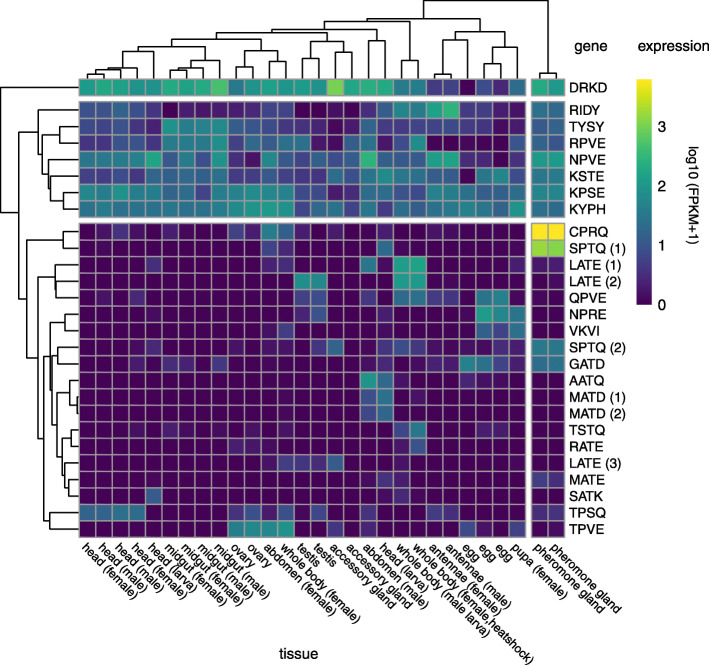
Expression profiling of FAD genes in different tissues of *Cydia pomonella*. The heatmap represents the absolute expression value (log FPKM) of all FAD genes in the corresponding tissues. Genes are identified by their signature motif. Automatic hierarchical clustering of FAD genes distinguishes three clusters: clusters I and II contain genes with ubiquitous expression across tissues and developmental stages; cluster III contains genes with more divergent expression and higher tissue specificity. Automatic hierarchical clustering of tissues indicates that pheromone gland samples have a distinguishable profile, which is characterized by the overexpression of Cpo_CPRQ and Cpo_SPTQ(1). Estimates of abundance values were obtained by mapping reads against the genome. The NCBI SRA accession numbers of all RNA-Seq data sets used are given in Additional file 6: Table S4

We found strong qualitative differences in the transcription profile of the 27 examined FAD genes (Fig. 5). The genes divide into three clusters. The first two correspond to genes that appear ubiquitously expressed. These include the two putative Front-End desaturases Cpo_DRKD and Cpo_RIDY (Cyt-b5-r), the Sphingolipid ∆4 desaturase Cpo_TYSY (Ifc), and five First desaturases, namely Cpo_NPVE (A1; ∆9 C16<C18), Cpo_KPSE (A1; ∆9 C16>C18), Cpo_RPVE (A1; ∆11/∆10), Cpo_KSTE (D), and Cpo_KYPH (E). With the exception of Cpo_RPVE, these genes exhibit a high evolutionary stability characterized by a single-copy gene with rare cases of gains and losses across insects. Their broad expression profiles suggest that these genes fulfill a fundamental metabolic function, making them unlikely candidates for pheromone biosynthesis. By contrast, the other First Desaturase genes found in the third cluster are characterized by a sparse expression profile and greater tissue and/or life-stage specificity: head: Cpo_TPSQ (A1; ∆11/∆10); ovary/female abdomen: Cpo_TPVE (B; ∆5); pheromone gland: Cpo_CPRQ (A1; ∆11/∆10), Cpo_SPTQ(1) (A1; ∆11/∆10); and eggs: Cpo_NPRE (A1), Cpo_VKVI (B; ∆6). These expression patterns are suggestive of more specialized functions. Specifically, their expression profiles and levels indicated that Cpo_CPRQ and Cpo_SPTQ(1) (average FPKM in pheromone gland, 6082.0 and 1451.5, respectively) represent primary candidates for functional testing.

### Functional characterization demonstrates the E9 FAD activity of Cpo_CPRQ and Cpo_SPTQ(1)

Previous studies have shown that the baker’s yeast *Saccharomyces cerevisiae* is a convenient system for studying in vivo the function of FAD genes and it has become the eukaryotic host of choice for the heterologous expression of desaturases from diverse sources including moths. Conveniently, yeast cells readily incorporate exogenous fatty acid methyl esters and convert them to the appropriate coenzyme A thioester substrates required by FADs.

**Fig. 6:**
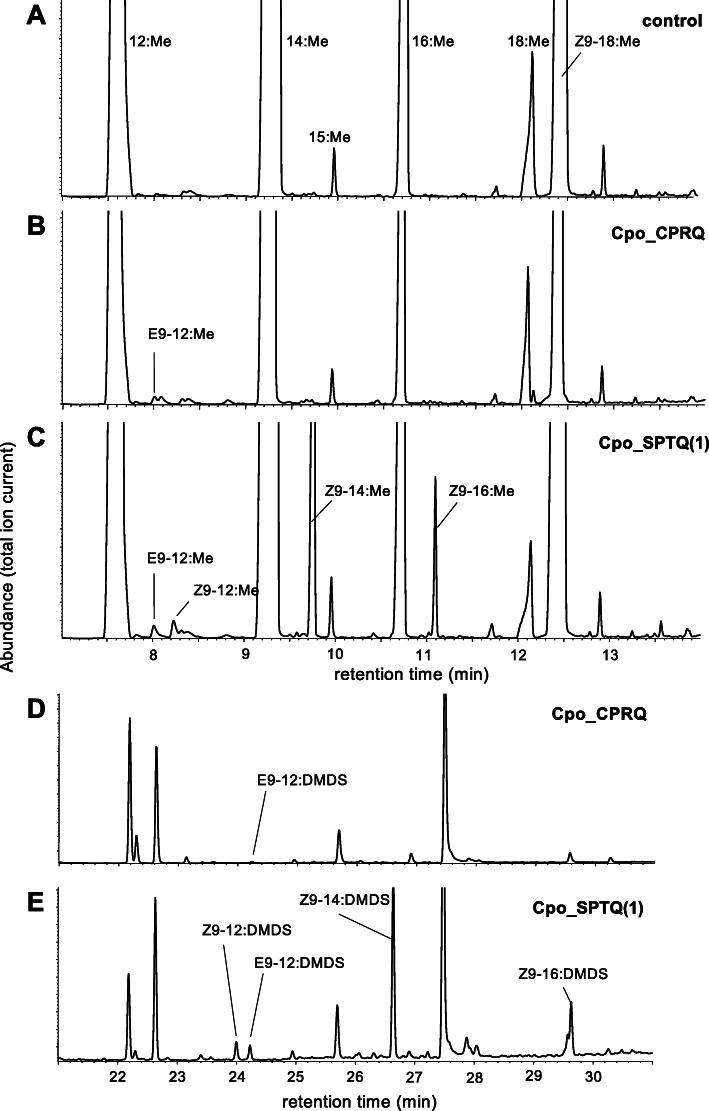
Functional characterization of desaturase activity of candidate genes in yeast. Total ion chromatograms of fatty acid methyl ester (FAME) products of Cu^2+^-induced *ole1 elo1 S. cerevisiae* yeast supplemented with saturated acyl precursors and transformed with **a** empty expression vector (control), **b** pYEX-CHT-Cpo_CPRQ, and **c** pYEX-CHT-Cpo_SPTQ(1). Confirmation of the identity of enzyme products was obtained by comparison of retention time and mass spectra of synthetic standards. Confirmation of double-bond positions by mass spectra of DMDS adducts (**d**, **e**)

First, we cloned Cpo_CPRQ and Cpo_SPTQ(1) into a copper-inducible yeast expression vector to generate heterologous expression in *S. cerevisiae*. We carried out assays with precursor supplementation to ensure availably of the medium-chain saturated fatty acids, i.e., lauric (C_12_) and myristic acids (C_14_), which are typically less abundant. Cpo_CPRQ conferred on the yeast the ability to produce a small amount of E9-12:Acyl and had no detectable activity outside of the C_12_ substrate (Fig. 6). Cpo_SPTQ(1) showed ∆9 desaturase activity, producing E9- and Z9-12:Acyl, Z9-14:Acyl, and Z9-16:Acyl. Double-bond positions in these products were confirmed by dimethyl disulfide (DMDS) derivatization (Fig. 6). In addition to retention times matching those of synthetic standards, spectra of the DMDS derivatives of the methyl esters displayed the following diagnostic ions: Z9-16:Me (M^+^, *m*/*z* 362; A^+^, *m*/*z* 145; B^+^, *m*/*z* 217), Z9-14:Me (M^+^, *m*/*z* 334; A^+^, *m*/*z* 117; B^+^, *m*/*z* 217), and E9- and Z9-12:Me (M^+^, *m*/*z* 306; A^+^, *m*/*z* 89; B^+^, *m*/*z* 217). No doubly unsaturated products were detected in these experiments.

In a second round of experiments, we assayed 7 other First desaturases that could be amplified from pheromone gland cDNA. As predicted from their respective placement in the FAD phylogeny, Cpo_NPVE, Cpo_KPSE, and Cpo_GATD encode ∆9 desaturases (Additional file 2: Figure S2). Cpo_NPVE is a ∆9 desaturase with preference for myristic (C_14_) and palmitic (C_16_) acid, showing only weak activity on lauric acid (C_12_). Cpo_GATD and Cpo_KPSE showed ∆9 desaturase activities mainly on C_16_ but also some activity on C_14_. The other 4 desaturases had no detectable activity in the yeast system.

Finally, we supplemented the growth medium with the monounsaturated methyl esters E9-12:Me and Z9-12:Me. The doubly unsaturated codlemone precursor, E8E10-12:Acyl, was inconsistently detected in some replicates of the assays with Cpo_CPRQ. No doubly unsaturated products were detected with the other constructs.

### Functional analysis identifies Cpo_CPRQ as the FAD catalyzing ∆8∆10 desaturation

**Fig. 7:**
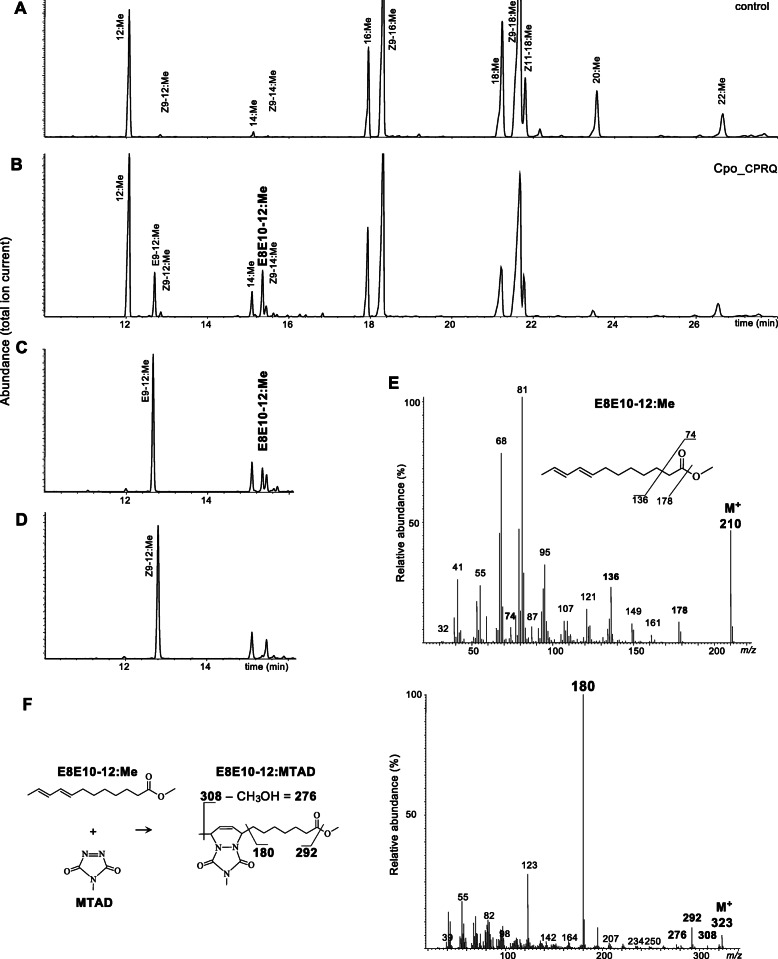
Functional characterization of desaturase activity of *Cpo_CPRQ* in insect cells. Total ion chromatograms of methyl esters (FAME) samples from Sf9 cells supplemented with lauric methyl ester (C_12_) infected with **a** empty virus (control) or **b** recombinant baculovirus expressing Cpo_CPRQ. Cpo_CPRQ produces large amount of E9-12:Me and E8E10-12:Me. Sf9 insect cells infected with bacmid expressing Cpo_CPRQ in the medium in the presence of the monoenic intermediate **c** (*E*)-9-dodecenoic methyl ester (E9-12:Me) or **d** (*Z*)-9-dodecenoic methyl ester (Z9-12:Me). The retention time and mass spectrum of the E8E10-12:Me peaks observed after addition of 12:Me and E9-12:Me were identical with those of the synthetic standard. The relatively long retention time (compound eluting later than 14:Me) is in agreement with what is expected from a diene with conjugated double bonds. **e** Spectrum of the E8E10-12:Me peak in **b**. The relatively abundant molecular ion *m*/*z* 210 is in agreement with the expectation for a diene with conjugated double bonds. **f** Analyses of MTAD-derivatized samples displayed diagnostic ions at *m/z* 323 (M^+^), *m/z* 308, and *m/z* 180 (base peak), confirming the identification of the conjugated double-bond system

In order to evaluate the presumptive ∆8∆10 desaturase activity of Cpo_CPRQ, we further investigated the activity of the enzyme in an insect cell line. We reasoned that the inconsistent activity of the enzyme in yeast could be due to the cellular environment of the expression system. When we expressed Cpo_CPRQ using the Sf9 cell system and supplementing the culture medium with lauric acid, we could observe the consistent production of E9-12 as well as E8E10-12, demonstrating that Cpo_CPRQ catalyzes the biosynthesis of E8E10-12:Acyl and its monounsaturated intermediate E9-12:Acyl (Fig. 7). As expected from the previous experiments in yeast, we did not detect activity on longer chain substrates. The retention time and mass spectrum of the E8E10-12:Me peak were identical to those of the synthetic standard. Double-bond positions of the conjugated system were confirmed by derivatization with 4-methyl-1,2,4-triazoline-3,5-dione (MTAD) (Fig. 7). Finally, we carried out assays to further evaluate the stereospecificity of the enzyme. When provided with E9-12:Me, Cpo_CPRQ produced the doubly unsaturated product (Fig. 7). In contrast, we found no evidence for activity on the corresponding diastereomer, Z9-12:Me (Fig. 7).

Altogether, these results show that Cpo_CPRQ exhibit both the ability to catalyze the *trans* desaturation of lauric acid to form E9-12:Acyl and the 1,4-desaturation of the latter to form the E8E10-12 conjugated pheromone precursor of codlemone.

## Discussion

In this study, we show the key role of a desaturase with a dual function in the biosynthesis of codlemone. We confirm the results suggested by the labeling experiments reported by Löfstedt and Bengtsson [31] and demonstrate that Cpo_CPRQ can conduct both the initial desaturation of 12:Acyl into E9-12:Acyl, and then transform E9-12:Acyl into E8E10-12:Acyl, the immediate fatty acyl precursor of E8E10-12:OH, the sex pheromone of *C. pomonella* (Fig. 2). Phylogenetically, Cpo_CPRQ clusters in the Lepidoptera-specific XXXQ/E clade (Fig. 4), a lineage which encodes pheromone biosynthetic enzymes with diverse properties [34]. Several FADs found in that clade have been demonstrated to be involved in the pheromone biosynthesis of many moth species, including the species of Tortricinae using ∆11 (*Epiphyas postvittana*, *Choristoneura rosaceana*, *Agryrotaenia velutinana*) and ∆10 desaturations (*Planotortrix octo*) [20–22, 40]. Our phylogenetic reconstruction indicates that Cpo_CPRQ does not appear orthologous to any of those genes (Additional file 1: Figure S1), suggesting the recruitment of a different ancestral duplicate early in the evolution of Olethreutinae. In addition to Cpo_CPRQ, Cpo_SPTQ(1) clustered in the same clade and had a similar activity profile on lauric acid, although it produces more Z9- than E9-12:Acyl. With an expression at about 25% the level of Cpo_CPRQ, Cpo_SPTQ(1) is the second most highly expressed FAD gene in the pheromone gland, a tissue in which this FAD appears exclusively expressed. Although Cpo_CPRQ appears sufficient to produce E8E10-12:Acyl, we cannot exclude the possibility that Cpo_SPTQ(1) contributes to the production of the monoene intermediate and is involved in the pheromone biosynthesis of codlemone or E9-12:OH, a pheromone component eliciting antennal response but with no apparent behavioral effect [45]. Interestingly, even though they are phylogenetically clustered within the ∆11/∆10-desaturase subfamily, Cpo_CPRQ and Cpo_SPTQ(1) exhibit primarily ∆9-desaturase activity, which likely represent the ancestral state. Whether this represents a conservation or a reversal to the ancestral function awaits further investigations. This finding provides exciting opportunities to study the structure-function relationships in FADs, in particular the structural determinant of regioselectivity.

Expansion of gene families is usually regarded as playing a key role in contributing to phenotypic diversity. The acyl-CoA desaturase gene family is characterized by a highly dynamic evolutionary history in insects [13, 14, 34]. Bursts of gene duplication have provided many opportunities for evolution to explore protein space and generate proteins with unusual and novel functions. In moths, many examples highlight the role played by the diversification in FAD functions in allowing the expansion of the multi-dimensional chemical space available for communication via sex pheromones. Careful re-annotation of the *C. pomonella* genome and phylogenetic analyses of identified desaturase genes allowed us to identify 27 genes representative of all desaturase subfamilies previously identified in insects. Interestingly, all functional classes previously identified in moths (i.e., ∆5, ∆6, ∆9, ∆10, ∆11, ∆14) have homologs in *C. pomonella*. This indicates that FAD genes tend to be preserved in the genomes for long periods of time. The availability of several chromosome-level assemblies in Lepidoptera has revealed that the genome architecture is generally conserved, even among distant species, with few to no structural rearrangements, i.e., inversions and translocations, being observed (but see [46]). This is attested by the high level of synteny existing between *C. pomonella* and *Spodoptera litura* (Lepidoptera: Noctuidae), two representatives of lineages which shared their last common ancestor ca. 120 MYA [33, 47]. This stability over long evolutionary time means that we can draw tentative conclusions from the genomic organization of gene families. Specifically, the genomic location of FAD genes in *C. pomonella* suggests that the diversification of this gene family is ancient. First Desaturase genes from all groups sensu Helmkampf et al. [14] have representatives in species that have shared their last common ancestor over 300 MYA. Moreover, we found several expansions with differing patterns within ∆9 C16>C18 (KPSE) and the Lepidoptera-specific gene subfamily ∆11, ∆10, and bifunctional (XXXQ/E). Genes clustered in tandem arrays represent evolutionary recent duplications, as exemplified by the expansion in ∆9 C16>C18 (KPSE). These duplicates are likely the product of imperfect homologous recombination. By contrast, with 9 representatives spread across 6 chromosomes, the ∆11/∆10 subfamily comprises both recent duplicates (e.g., Cpo_SPTQ(1) and Cpo_SPTQ(2)) as well as unlinked FAD genes (such as Cpo_CPRQ). The latter genes are likely to be the products of more ancient events of duplications and chromosomal rearrangements. The location of the majority of genes within this clade suggests that the diversification within this subfamily essential for the pheromone biosynthesis occurred early in the evolution of the Lepidoptera. As genome data from more species become available, it will be possible to follow the evolutionary trajectories of this gene family which has been a key contributor to pheromone evolution.

Expansion of gene families is often followed by a relaxation of selection, which translates into higher rates of fixation of mutations, including non-synonymous nucleotide substitutions [48, 49]. Looking at the impact of selection on desaturase genes in insects, previous studies found little evidence for positive selection but rather purifying selection acting on all desaturase genes in insects, even in expanded subfamilies [14, 34]. This could be indicative that large constraints along the coding sequences to not change sites affecting the proper enzymatic function outweigh the potential for diversifying selection. Desaturases encoded by genes in the ∆11/∆10 (XXXQ/E) lineages display a great variety of enzymatic functions, which are paralleled by very high levels of sequence divergence. The lack of evidence for positive selection could result from a lack of power in the statistical analysis currently available under when analyzing genes selected for very diverse properties [34]. Modification of the regulation of duplicated genes is also expected, with some paralogs specializing to a limited set of tissues or developmental stages [49]. Data from a panel representing a relatively large variety of tissue types and developmental stages reveal a large expression divergence between FAD genes. Interestingly, we see that genes present in the recent expansion (i.e., ∆9 C16>C18 (KPSE)), of which members form tandem arrays in the genome, have low to no expression in the large panel of samples we analyzed. The evidence at hand suggests that these may have limited physiological relevance. This advocates for caution when interpreting the importance and functional consequences of expansions. Some expansions can contribute to lineage-specific adaptation and an increased demand for variability in associated traits. However, it is almost certain that many expansions will not reflect a response to changes and be responsible for the evolution of novel traits but rather have their evolutionary origin in the stochastic nature of the gene duplication process. Functional testing is paramount to advance our mechanistic understanding of the proximate consequences of variation in the size of multigene families.

Previously characterized FADs that catalyze the formation of conjugated fatty acids among moth sex pheromones precursors fall into two groups: (1) double bonds can be inserted via sequential 1,2-desaturation to produce a diene, either by the action of two different desaturases operating consecutively as postulated in *Dendrolimus punctatus* (Lasiocampidae) [42], or by the same desaturase operating twice with an intermediate chain-shortening step as suggested for *Epiphyas postvittana* (Tortricidae) [20], *L. capitella* [41], and *Spodoptera litura* (Noctuidae) [50]; (2) bifunctional desaturases can introduce the first double bond and then rearrange the monoene to produce a diene with conjugated double bonds via 1,4-desaturation, as seen in *Bombyx mori* (Bombycidae) [43], *S. littoralis* (Noctuidae) [51], and *Manduca sexta* (Sphingidae) [44]. The *C. pomonella* desaturase falls into the latter category: Cpo_CPRQ introduces first an *E*9 double bond in the 12:Acyl saturated substrate and then transforms the *E*9 unsaturation into the *E*8*E*10 conjugated double bonds. The independent evolution of similar features in enzymes used by distantly related species offers the opportunity to further study mechanistically the determinants of the switch between 1,2 and 1,4-dehydrogenation.

**Fig. 8:**
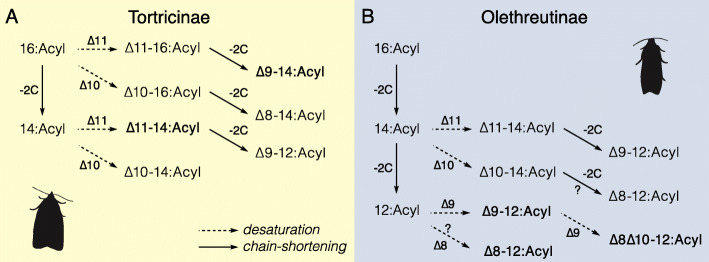
Postulated biosynthetic steps to account for the typical pheromone components found in the Tortricidae. Fatty acid biosynthetic routes for sex pheromone components of Tortricinae (**a**) and Olethreutinae (**b**) are illustrated. Starting from palmitic acyl (C_16_), the pheromone precursors are produced via a combination of chain-shortening (by limited beta-oxidation) and desaturation steps. Genes encoding the fatty acyl desaturases with the postulated activities have been characterized in this study or previously, with the exception of the enzyme(s) involved in the synthesis of ∆8 monoenes in Olethreutinae

Recent molecular phylogenetic studies [18, 19] provide strong support for the monophyly of Tortricinae and Olethreutinae, the two most speciose subfamilies within Tortricidae, and the paraphyly of Chlidanotinae. Our tribal-level tree for Tortricidae combined with data on moth pheromones and attractants corroborate the pattern of pheromone components in Tortricinae and Olethreutinae reviewed and discussed by Roelofs and Brown [17]. Tortricinae species use mostly 14-carbon pheromone components with unsaturation introduced via ∆11 desaturation (Fig. 8). Molecular and biochemical studies have confirmed the paramount role of ∆11 FADs in the pheromone biosynthesis of not only tortricines, but the vast majority of ditrysian Lepidoptera (see for instance [13, 41, 52–54]). No pheromones are reported for the Chlidanotinae. However, the sex attractants reported for three species include Z9-14:OAc and Z11-16:OAc, which supports the idea that ∆11 desaturation in combination with chain-shortening may be typical of this subfamily. By contrast, the Olethreutinae subfamily uses mainly 12-carbon compounds. The ∆9-12 chain structures used by many species could be accounted for the action of a ∆11 enzyme producing ∆11-14:Acyl as an intermediate following by chain-shortening to ∆9-12:Acyl (Fig. 8). For example, the host races of the larch budworm *Zeiraphera diniana* (Tortricidae: Olethreutinae: Eucosmini) developing on larch (*Larix decidua*) or Cembra pine (*Pinus cembra*) used respectively E11-14:OAc and E9-12:OAc as pheromone (Guerin et al. 1984), and it is parsimonious to hypothesize that polymorphism in the activity of the chain-shortening enzyme is responsible for this difference between host races that show little to moderate genomic differentiation [55]. Our data suggest however that an alternative pathway may exist. Indeed, two of the enzymes characterized in the present study (i.e., Cpo_SPTQ(1) and Cpo_CPRQ) can catalyze the production of ∆9-12:Acyl directly from lauric acid (C_12_), bypassing the need for prior ∆11 desaturation and chain-shortening (Fig. 8).

In addition to ∆9-12 chain structures, 12-carbon components with ∆8 and ∆8∆10 systems are frequently used by olethreutines. Specifically, these occur in the three major tribes: Grapholitini, Eucosmini, and Olethreutini (Fig. 1). The identification of the bifunctional desaturase catalyzing the biosynthesis of ∆8∆10-12 fatty acid derivatives sheds new light on the pathway associated with the production of pheromones of a large number of tortricids. Monounsaturated compounds with double bonds in ∆8 position frequently co-occur with conjugated ∆8∆10, including in trace amount in *C. pomonella* [45]. However, since we did not detect any ∆8 activity towards the lauric acid from Cpo_CPRQ or Cpo_SPTQ(1), we anticipate that a different FAD plays a role in the pheromone biosynthesis of ∆8 monoenes. This is supported by the pheromone bouquets identified for several species of olethreutines. In the podborer *Matsumuraeses falcana* (Tortricidae: Olethreutinae: Grapholitini), the pheromone consists of a mixture of E8-12:OAc and E8E10-12:OAc, plus minor amounts of (*E*7,*Z*9)- and (*E*7,*E*9)-dodecadienyl isomers, and the monoenes E10-12:OAc and E10-14:OAc [56]. The major pheromone component in *Hedya nubiferana* (Tortricidae: Olethreutinae: Olethreutini) is E8E10-12:OAc, but in addition, the pheromone contains relatively large amounts of Z8- and E8-12:OAc [57]. The same holds for *Epiblema foenella* (Tortricidae: Olethreutinae: Eucosmini) in which the major pheromone component was identified as Z8Z10-12:OAc: in addition to the other three geometric isomers of the diene, the females produce also E8- and Z8-12:OAc but no E9-12:OAc [32]. Biosynthesis of these bouquets suggests the involvement of a FAD with ∆8 or ∆10 activity (Fig. 8). A ∆10 FAD is the key enzyme involved in the production of monounsaturated compounds with double bonds in ∆8 position. Specifically, in *Planotortrix* sp. and *Ctenopseustis* sp. (Tortricidae: Tortricinae: Archipini), Z8-14:OAc is biosynthesized via ∆10 desaturation of palmitic acid (C_16_) followed by chain-shortening, whereas the production of Z10-14:OAc involves the same FAD acting on myristic acid (C_14_) [12, 40, 58] (Fig. 8a). Our phylogenetic analysis of FAD genes shows that the *C. pomonella* genome contains several genes clustering with the tortricine ∆10 FADs, indicating that this biochemical activity may in theory still be carried out in olethreutines. Additional work is necessary to elucidate the routes towards the biosynthesis of monounsaturated pheromone components with double bond at even positions.

On a practical point of view, pheromones have gradually been added to the pest control toolkit. In the specific case of *Cydia pomonella*, in 2010, the annual production of synthetic codlemone—the codling moth pheromone—was estimated at 25 metric tons, which were used for the treatment by mating disruption of 200,000 ha of orchards worldwide [59]. The biological production of moth pheromones in plants or microorganisms such as yeast has been suggested as an environmentally friendly alternative to conventional chemical synthesis [60]. Beyond the stage of conceptualization, the approach is proven, and large-scale production is on the horizon [61–64]. Identification and characterization of the key enzymes underlying production of pheromone is a crucial step to assemble the building blocks central to the engineering of plant and cell factories. In this context, the identification and characterization of a desaturase involved in pheromone biosynthesis in *C. pomonella* is of particular interest, as it represents an important step towards the biological production of pheromone for the integrated pest management of several economically important olethreutines.

## Conclusions

The fatty acid desaturase gene family is central to the pheromone-based communication of many insects. Our integrative approach shows that the evolution of the signature pheromone structure of olethreutine moths involved the recruitment of a gene resulting from an ancient gene duplication within a Lepidoptera-specific desaturase lineage. Members of other expanded FAD subfamilies do not appear to play a role in chemical communication. This advises for caution when postulating the consequences of lineage-specific expansions based on genomics alone.

## Methods

### Semiochemical data set

To compile a dataset of the semiochemicals used as sex attractants by tortricid moths, we manually retrieved data from the Pherobase, a freely accessible database of pheromones and semiochemicals which compiles information from the literature and peer-reviewed publications [65]. Altogether, we gathered data for 536 taxa belonging to the family Tortricidae. Specifically, we were interested in collecting information about the chemical structure of the bioactive fatty acyl derivatives, i.e., double-bond position and number and length of the aliphatic chain. Following the database convention, we classified bioactive compounds as *pheromone* or *attractant*. The term pheromone denotes a component of the female sex attractant, identified from females, and with demonstrated biological activity; attractant characterizes those bioactive compounds found by male responses alone, such as by systematic field screening or by significant non-target capture in traps baited with pheromone lures. Although these compounds are not necessarily used in the natural communication system, they have in most cases a high probability of being pheromone components. Details are in Additional file 3: Table S1.

### Molecular data set

To infer the evolutionary relationship among the species represented in our trait dataset, we used sequence data from 137 terminal taxa in our phylogenetic analysis, including 131 species of Tortricidae as ingroup. The outgroup contained six taxa, each a species representative of Cossidae, Galacticidae, Heliocosmidae, Lacturidae, Limacodidae, and Sesiidae, respectively. Our data set is based largely on Regier et al. [18] and Fagua et al. [19], augmented with a few additional Genbank accessions. Following the aforementioned authors, we used regions of five nuclear genes and one mitochondrial gene. The nuclear genes were *carbamoyl-phosphate synthetase II* (CAD), *dopa decarboxylase* (DDC), *enolase* (Eno), *period* (PER), and *wingless* (WG); the mitochondrial gene was the fragment of *cytochrome oxidase I* (COI) used for barcoding. Details are in Additional file 4: Table S2. For each genus, we used one representative species as the type species. Our main goal was to place the major transition in the pheromone communication system of Tortricidae and thus to infer the phylogenetic relationships with good resolution at the subfamily and tribe level. We retrieved a total of 340 Genbank accessions (see Additional file 4: Table S2 for accession numbers).

### Phylogenetic tree

We concatenated the molecular data corresponding to one taxon and obtained an alignment of the entire molecular data set using MAFFT [66] as implemented in Geneious (Biomatters). The final alignment comprised 7591 sites. To define the best scheme of partitions using the MAFFT alignment, we used PartitionFinder v2.1.1 [67]. We tested models of nucleotide substitution with partitions representing codon position and genes using *greedy* search [68], *AICC* model selection, and setting the phylogeny program to *phyml* [69] (default value for all other settings). A 16-partition scheme was selected and used to perform the parametric phylogenetic analysis in IQ-TREE 1.6.11 [70, 71]. The maximum likelihood analysis was performed using default settings and by calculating Shimodaira-Hasegawa-like approximate ratio test (SH-aLRT) support and ultrafast bootstrap support (UFBoot [72];) after 1000 replicates each. We used the R package *ggtree* [73] for visualizing and annotating the phylogenetic tree with associated semiochemical data.

### Insects, tissue collection, and RNA-Seq

Pupae of *C. pomonella* were purchased from Entomos AG (Switzerland) and were sexed upon arrival to our laboratory. Adult males and females emerged in separate jars in a climate chamber at 23 ± 1 °C under a 16 h:8 h light to dark cycle. On the third day after emergence, pheromone glands of female moths were dissected, immersed in TRIzol Reagent (Thermo Scientific), and stored at − 80 °C until RNA extraction. Two pools of 30 glands were collected 3 h before and after the onset of the photophase, respectively. Total RNA was extracted according to the manufacturer’s instructions. RNA concentration and purity were initially assessed on a NanoDrop2000 (Thermo Fisher Scientific). Library preparation and paired-end Illumina sequencing (2 × 100 bp) were performed by BGI (Hong Kong, PRC). We obtained ~ 66 million QC-passing reads per sample.

### Genome annotation improvements

To ensure that all FAD genes were correctly annotated in the *C. pomonella* genome, we used our RNA-Seq data of pheromone gland to improve the annotation (cpom.ogs.v1.chr.gff3) which we obtained from InsectBase (http://www.insect-genome.com/cydia/). We performed low-quality base trimming and adaptor removal using cutadapt version 1.13 [74] and aligned the trimmed read pairs against the genome using HISAT2 version 2.2.0 [75]. The existing annotation was used to create a list of known splice sites using a python script distributed with HISAT2. We used StringTie version 2.1.3b [76] with the *conservative* transcript assembly setting to improve the annotation and reconstruct a non-redundant set of transcripts observed in any of the RNA-Seq samples. We applied Trinotate version 3.2.1 [77] to generate a functional annotation of the transcriptome data. We identified candidate FAD genes by searching for genes harboring the PFAM domain corresponding to a fatty acid desaturase type 1 domain (PF00487) in their predicted protein sequences. We used the R package *KaryoploteR* [78] to visualize the location of the FAD genes.

### Desaturase phylogenetic analysis

To investigate the evolutionary relationship of the *C. pomonella* FADs, we carried out a phylogenetic analysis with other moth FAD proteins. Our sampling includes all the functional classes previously identified in moths (i.e., ∆5, ∆6, ∆9, ∆10, ∆11, ∆14) and representatives of Tortricidae as well as other moth families (see Additional file 5: Table S3 for list of taxa). Protein sequences for these enzymes were downloaded from GenBank. Protein sequences for *C. pomonella* were predicted from the genome using gffread [79] with our improved annotation.

We aligned amino acid sequences using MAFFT version 7.427 [66, 80]. We used the L-INS-i procedure and the BLOSUM30 matrix as the scoring matrix. The final multiple sequence alignment contained 114 sequences with 805 amino acid sites. The phylogenetic tree was constructed in IQ-TREE 1.6.11 [70]. An automatic model search was performed using ModelFInder [81] with the search restricted to models including the WAG, LG, and JTT substitution models. The maximum likelihood analysis was performed using default settings and by calculating Shimodaira-Hasegawa-like approximate ratio test (SH-aLRT) support and ultrafast bootstrap support (UFBoot [72];) after 1000 replicates each. We used the R package *ggtree* [73] for visualizing and annotating the phylogenetic tree, with midpoint rooting performed using the midpoint.root function implemented in the *phytools* package [82].

### Transcript abundance estimation

In addition to our pheromone gland data sets, we retrieved 27 already published Illumina paired-end RNA-Seq data sets for *C. pomonella* available on the Sequence Read Archive (SRA) (see Additional file 6: Table S4 for accession numbers). These data correspond to various tissues or life stages. Following QC, low-quality base trimming, and adaptor removal with cutadapt, read pairs were aligned against the genome using HISAT2. The obtained alignment BAM files were used to estimate transcript abundance using StringTie together with our improved annotation. The abundance tables from StringTie were imported into R using the *tximport* package [83], which was used to compute gene-level abundance estimates reported as FPKM. We used the R package *pheatmap* [84] to visualize the expression level of FAD genes.

### Cloning of desaturases

First-strand cDNAs were synthesized from 1 μg of total RNA using the SuperScript IV cDNA synthesis kit (Thermo Scientific). The cDNA products were diluted to 50 μL. PCR amplification (50 μL) was performed using 2.5 μL of cDNA as template with *attB*-flanked gene-specific primers (Additional file 7: Table S5) in a Veriti Thermo Cycler (Thermo Fisher Scientific) using Phusion polymerase master mix (Thermo Fisher Scientific). Cycling parameters were as follows: an initial denaturing step at 94 °C for 5 min, 35 cycles at 96 °C for 15 s, 55 °C for 30 s, 72 °C for 1 min followed by a final extension step at 72 °C for 10 min. Specific PCR products were cloned into the pDONR221 vector (Thermo Fisher Scientific) in the presence of BP clonase (Invitrogen). Plasmid DNAs were isolated according to standard protocols and recombinant plasmids were subjected to sequencing using universal M13 primers and the BigDye terminator cycle sequencing kit v1.1 (Thermo Fisher Scientific). Sequencing products were EDTA/ethanol-precipitated, dissolved in formamide, and loaded for analysis on an in-house capillary 3130xl Genetic analyzer (Applied Biosystems).

### Pheromone compounds and corresponding fatty acid precursors

All synthetic pheromone compounds used as references for identification came from our laboratory collection unless otherwise indicated. Pheromone compounds are referred to as short forms, e.g., (*E*8,*E*10)-8,10-dodecadienol is E8E10-12:OH, the corresponding acetate would be E8E10-12:OAc, corresponding acyl moiety (dodecadienoate) is E8E10-12:Acyl (although it may occur as CoA-derivative), and the methyl ester thereof is referred to as E8E10-12:Me. The dodecanoic methyl ester (methyl laurate, 12:Me) and tetradecanoic methyl ester (methyl myristate, 14:Me) were purchased from Larodan Fine Chemicals (Malmö, Sweden). The (*E*)-9-dodecenol (E9-12:OH) was purchased from Pherobank (Wageningen, The Netherlands) and then converted into the corresponding methyl ester (E9-12:Me). E8E10-12:OAc was purchased from Bedoukian (Danbury, CT, USA) and converted to the corresponding alcohol by hydrolysis using a 0.5-M solution of KOH in methanol. Fatty alcohols were oxidized to the corresponding acid with pyridinium dichromate in dimethylformamide as described by Bjostad and Roelofs [85]. The methyl esters were prepared as described under fatty acid analysis (see below).

### Heterologous expression of desaturases in yeast

Plasmids containing the genes of interest were selected as entry clones and subcloned into the copper-inducible pYEX-CHT vector [86]. Sequences of recombinant constructs were verified by Sanger sequencing. The pYEX-CHT recombinant expression vectors harboring *C. pomonella* FADs were introduced into the double deficient *elo1∆*/*ole1*∆ strain (*MATa elo1::HIS3 ole1::LEU2 ade2 his3 leu2 ura3*) of the yeast *Saccharomyces cerevisiae*, which is defective in both desaturase and elongase functions [87] using the *S.c.* EasyComp Transformation kit (Thermo Fisher Scientific). For selection of uracil and leucine prototrophs, the transformed yeast was allowed to grow on an SC plate containing 0.7% YNB (w/o AA; with ammonium sulfate) and a complete drop-out medium lacking uracil and leucine (Formedium, UK), 2% glucose, 1% tergitol (type Nonidet NP-40, Sigma), 0.01% adenine (Sigma), and containing 0.5 mM oleic acid (Sigma) as extra fatty acid source. After 4 days at 30 °C, individual colonies were selected and used to inoculate 10 mL selective medium, which was grown at 30 °C at 300 rpm in a shaking incubator for 48 h. Yeast cultures were diluted to an OD_600_ of 0.4 in 10 mL of fresh selective medium containing 2 mM CuSO_4_ for induction, with or without addition of a biosynthetic precursor. Yeast cells contain sufficient quantities of naturally occurring palmitic acid; hence, supplementation with 16:Me was typically not necessary. Lauric acid and myristic acid were added to the medium as methyl esters (12:Me and 14:Me). In subsequent assays, the monounsaturated methyl esters E9-12:Me and Z9-12:Me were also supplemented. Each FAME precursor was prepared at a concentration of 100 mM in 96% ethanol and added to reach a final concentration of 0.5 mM in the culture medium. Yeasts were cultured in 30 °C with Cu^2+^ induction. After 48 h, yeast cells were harvested by centrifugation at 3000 rpm and the supernatant was discarded. The pellets were stored at − 80 °C until fatty acid analysis. Yeast expression experiments were conducted with three independent replicates.

### Heterologous expression of desaturases in insect cells

The expression construct for Cpo_CPRQ in the baculovirus expression vector system (BEVS) donor vector pDEST8_CPRQ was made by LR reaction. Recombinant bacmids were made according to instructions for the Bac-to-Bac® Baculovirus expression system given by the manufacturer (Invitrogen) using DH10EMBacY (Geneva Biotech). Baculovirus generation was done using *Spodoptera frugiperda* Sf9 cells (Thermo Fisher Scientific), Ex-Cell 420 serum-free medium (Sigma), and baculoFECTIN II (OET). The virus was then amplified once to generate a P2 virus stock using Sf9 cells and Ex-Cell 420 medium. Viral titer in the P2 stock was determined using the BaculoQUANT all-in-one qPCR kit (OET) and found to be 3 × 10^8^ pfu/mL for Cpo_CPRQ.

Insect cell lines Sf9 were diluted to 2 × 10^6^ cells/mL. Heterologous expression was performed in 20-mL cultures in Ex-Cell 420 medium and the cells infected at an MOI of 1. The cultures were incubated in 125-mL Erlenmeyer flasks (100 rpm, 27 °C), with fatty acid methyl-ester substrates supplemented at a final concentration of 0.25 mM after 1 day. After 3 days, 7.5-mL samples were taken from the culture and centrifuged for 15 min at 4500*g* at 4 °C. The pellets were stored at − 80 °C until fatty acid analysis. Sf9 expression experiments were conducted in three replicates.

### Fatty acid analysis

Total lipids from cell pellets were extracted using 3.75 mL of methanol/chloroform (2:1 v/v) in clear glass tubes. One milliliter of 0.15 M acetic acid and 1.25 mL of water were added to wash the chloroform phase. Tubes were vortexed vigorously and centrifuged at 2000 rpm for 2 min. The bottom chloroform phase (~ 1 mL), which contains the total lipids, was transferred to a new glass tube. Fatty acid methyl esters (FAMEs) were produced from this total lipid extract. The solvent was evaporated to dryness under gentle nitrogen flow. One milliliter of sulfuric acid 2% in methanol was added to the tube, vortexed vigorously, and incubated at 90 °C for 1 h. After incubation, 1 mL of water was added and mixed well, and 1 mL of hexane was used to extract the FAMEs.

The methyl ester samples were subjected to GC-MS analysis on a Hewlett Packard 6890 GC (Agilent) coupled to a mass selective detector HP 5973 (Agilent). The GC was equipped with an HP-INNOWax column (30 m × 0.25 mm × 0.25 μm; Agilent), and helium was used as carrier gas (average velocity 33 cm/s). The MS was operated in electron impact mode (70 eV), and the injector was configured in splitless mode at 220 °C. The oven temperature was set to 80 °C for 1 min, then increased at a rate of 10 °C/min up to 210 °C, followed by a hold at 210 °C for 15 min, and then increased at a rate of 10 °C/min up to 230 °C followed by a hold at 230 °C for 20 min. The methyl esters were identified based on mass spectra and retention times in comparison with those of our collection of synthetic standards.

Double-bond positions of monounsaturated compounds were confirmed by dimethyl disulfide (DMDS) derivatization [88], followed by GC-MS analysis. FAMEs (50 μL) were transferred to a new tube, 50 μL DMDS were added, and the mixture was incubated at 40 °C overnight in the presence of 5 μL of iodine (5% in diethyl ether) as catalyst. Hexane (200 μL) was added to the sample, and the reaction was neutralized by addition of 50–100 μL sodium thiosulfate (Na_2_S_2_O_3_; 5% in water). The organic phase was recovered and concentrated under a gentle nitrogen stream to a suitable volume [41]. DMDS adducts were analyzed on an Agilent 6890 GC system equipped with a HP-5MS capillary column (30 m × 0.25 mm × 0.25 μm; Agilent) coupled with an HP 5973 mass selective detector. The oven temperature was set at 80 °C for 1 min, raised to 140 °C at a rate of 20 °C/min, then to 250 °C at a rate of 4 °C/min and held for 20 min [36].

Double-bond positions of di-unsaturated compounds with conjugated double bonds were confirmed by derivatization with 4-methyl-1,2,4-triazoline-3,5-dione (MTAD) [89]. MTAD adducts were prepared using 15 μL of the methyl ester extracts transferred into a glass vial. The solvent was evaporated under gentle nitrogen flow, and 10 μL of dichloromethane (CH_2_Cl_2_) was added to dissolve the FAMEs. The resulting solution was treated with 10 μL of MTAD (Sigma; 2 mg/mL in CH_2_Cl_2_). The reaction was ran for 10 min at room temperature, and 2 μL was subjected to GC-MS analysis on an Agilent 6890 GC system equipped with a HP-5MS capillary column (30 m × 0.25 mm × 0.25 μm; Agilent) coupled with an HP 5973 mass selective detector. The injector temperature was set to 300 °C and the oven was set to 100 °C and then increased by 15 °C/min up to 300 °C followed by a hold at 300 °C for 20 min.

## Supplementary Information

**Additional file 1: Figure S1.** Phylogeny of lepidoptera FAD genes. Extended version of the maximum likelihood tree displayed in Fig. 4. The tree was obtained for predicted amino acid sequence of 114 FAD genes (805 aligned positions) of 28 species, with branch support values calculated from 1000 replicates using the Shimodaira-Hasegawa-like approximate ratio test (SH_aLRT) and ultrafast bootstrapping (UFboot). Support values for branches are indicated by colored circles, with color assigned based SH-aLRT and UFBoot supports with 80% and 95% as thresholds of branch selection for SH-aLRT and UFBoot supports, respectively. The major constituent six subfamilies of First Desaturase (A1 to E) and two subfamilies of Front-End (Cyt-b5-r) and Sphingolipid Desaturases (Ifc), respectively, are indicated following the nomenclature proposed by Helmkampf et al. (2015). For First Desaturases, the different shades correspond to the indicated putative biochemical activities and consensus signature motif (if any). Triangles indicate sequences from *C. pomonella*. The scale bar represents 0.5 substitutions per amino acid position. Species are indicated by three- or four-letter prefixes (see Additional file 5: Table S3 for details). Biochemical activities (or signature motif) are indicated after the abbreviated species name, followed by accession number in parenthesis.

**Additional file 2: Figure S2.** Functional characterization of desaturase activity of First-desaturases. Total ion chromatograms of fatty acid methyl ester (FAME) products of Cu^2+^-induced *ole1 elo1 S. cerevisae* yeast supplemented with saturated acyl precursors and transformed with (A & C) empty expression vector (control), (B) pYEX-CHT-Cpo_NPVE, (D) pYEX-CHT-Cpo_KPSE, and (E) pYEX-CHT-Cpo_GATD.

**Additional file 3: Table S1.** List of terminal taxa and corresponding bioactivity data used in this study.

**Additional file 4: Table S2.** List of terminal taxa and corresponding GenBank accession numbers used in this study.

**Additional file 5: Table S3.** List of species represented in the FAD phylogeny.

**Additional file 6: Table S4.** List of SRA accessions corresponding to RNA-Seq data sets for *Cydia pomonella*.

**Additional file 7: Table S5.** Primer sequences used for amplification and cloning of desaturase genes.

## Data Availability

All data generated or analyzed during this study are included in this published article and its supplementary information files.

## Abbreviations

FAD: Fatty acyl desaturase
FAME: Fatty acid methyl ester
DMDS: Dimethyl disulfide
MTAD: 4-Methyl-1,2,4-triazoline-3,5-dione
