# Supplementary figures and images for: Evolution of the codling moth pheromone via an ancient gene duplication
Source: BMC Biol. 2021 Apr 23;19:83. doi: 10.1186/s12915-021-01001-8 (PMC8063362; doi:10.1186/s12915-021-01001-8)

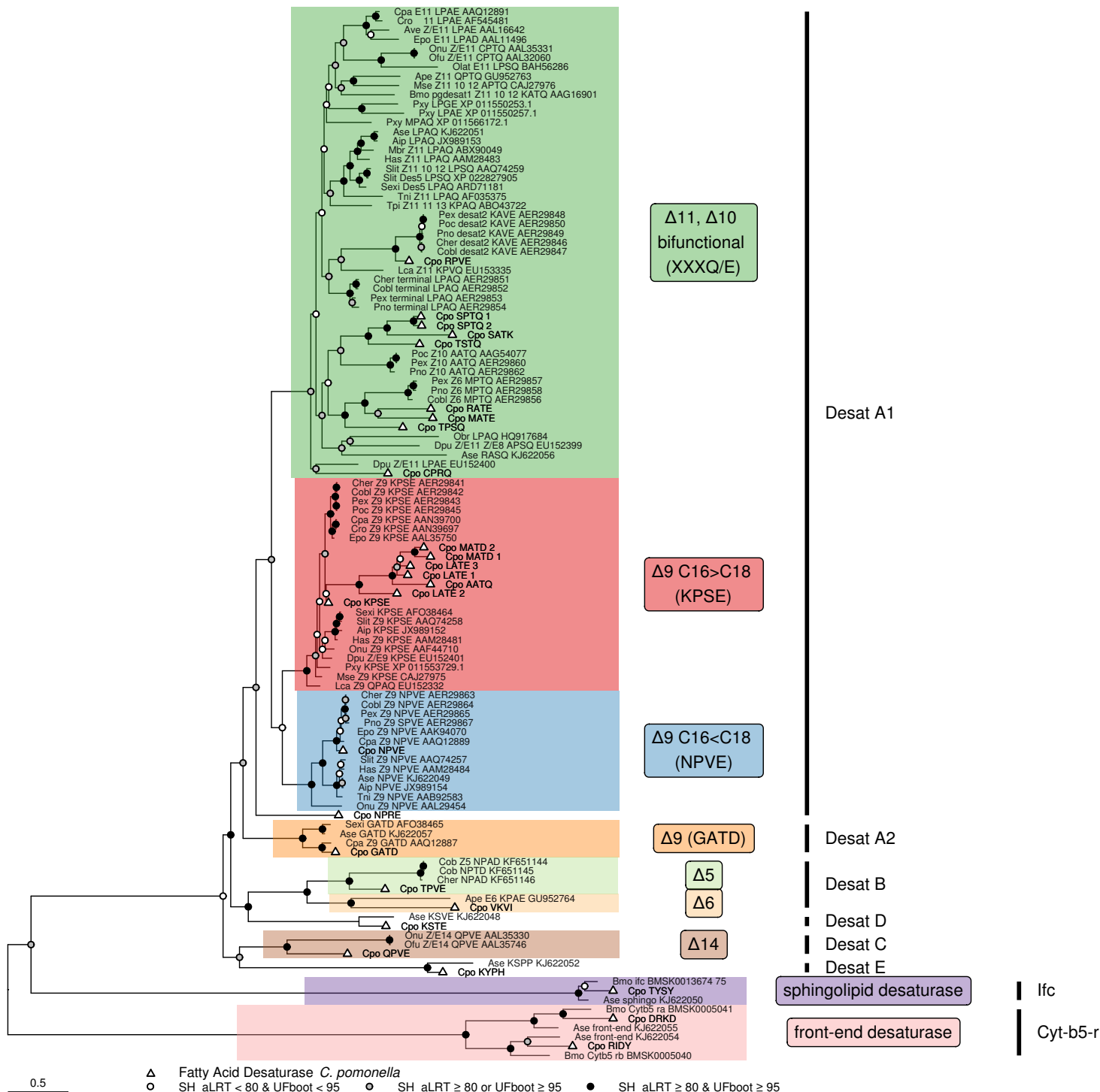

Supplement: Supplementary file 1 — Additional file 1: Figure S1. Phylogeny of lepidoptera FAD genes. Extended version of the maximum likelihood tree displayed in Fig. 4. The tree was obtained for predicted amino acid sequence of 114 FAD genes (805 aligned positions) of 28 species, with branch support values calculated from 1000 replicates using the Shimodaira-Hasegawa-like approximate ratio test (SH_aLRT) and ultrafast bootstrapping (UFboot). Support values for branches are indicated by colored circles, with color assigned based SH-aLRT and UFBoot supports with 80% and 95% as thresholds of branch selection for SH-aLRT and UFBoot supports, respectively. The major constituent six subfamilies of First Desaturase (A1 to E) and two subfamilies of Front-End (Cyt-b5-r) and Sphingolipid Desaturases (Ifc), respectively, are indicated following the nomenclature proposed by Helmkampf et al. (2015). For First Desaturases, the different shades correspond to the indicated putative biochemical activities and consensus signature motif (if any). Triangles indicate sequences from C. pomonella. The scale bar represents 0.5 substitutions per amino acid position. Species are indicated by three- or four-letter prefixes (see Additional file 5: Table S3 for details). Biochemical activities (or signature motif) are indicated after the abbreviated species name, followed by accession number in parenthesis. [file 12915_2021_1001_MOESM1_ESM.pdf]

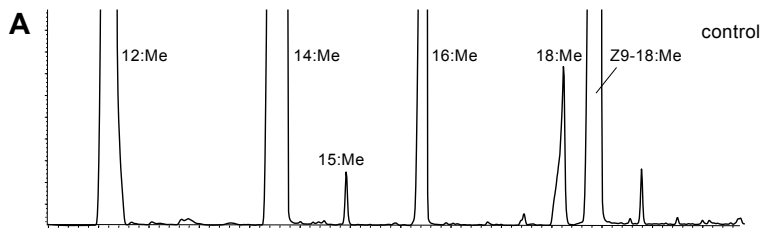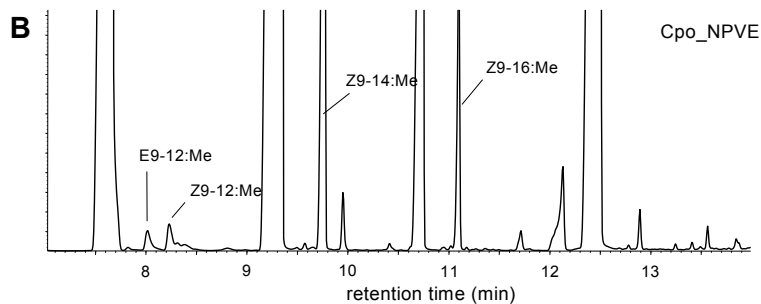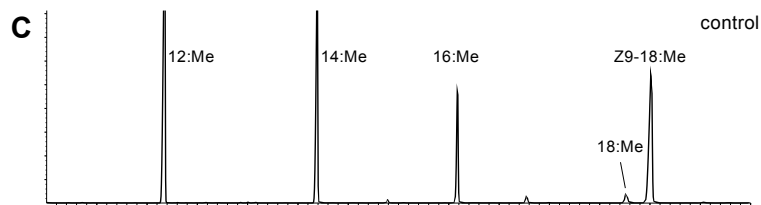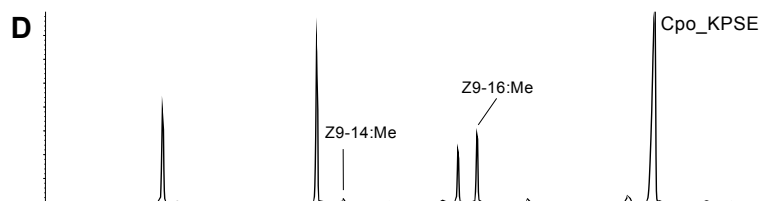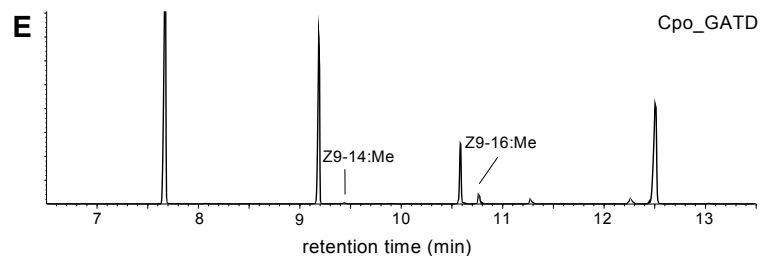

Supplement: Supplementary file 2 — Additional file 2: Figure S2. Functional characterization of desaturase activity of First-desaturases. Total ion chromatograms of fatty acid methyl ester (FAME) products of Cu2+-induced ole1 elo1 S. cerevisae yeast supplemented with saturated acyl precursors and transformed with (A & C) empty expression vector (control), (B) pYEX-CHT-Cpo_NPVE, (D) pYEX-CHT-Cpo_KPSE, and (E) pYEX-CHT-Cpo_GATD. [file 12915_2021_1001_MOESM2_ESM.pdf]
